# Functional Effects of EPS-Producing *Bifidobacterium* Administration on Energy Metabolic Alterations of Diet-Induced Obese Mice

**DOI:** 10.3389/fmicb.2019.01809

**Published:** 2019-08-07

**Authors:** Nuria Salazar, Audrey M. Neyrinck, Laure B. Bindels, Céline Druart, Patricia Ruas-Madiedo, Patrice D. Cani, Clara G. de los Reyes-Gavilán, Nathalie M. Delzenne

**Affiliations:** ^1^Metabolism and Nutrition Research Group, Louvain Drug Research Institute (LDRI), Université Catholique de Louvain (UCLouvain), Brussels, Belgium; ^2^Department of Microbiology and Biochemistry of Dairy Products, Instituto de Productos Lácteos de Asturias, Consejo Superior de Investigaciones Científicas (IPLA-CSIC), Asturias, Spain; ^3^Diet, Microbiota and Health Group, Instituto de Investigación Sanitaria del Principado de Asturias (ISPA), Oviedo, Spain; ^4^Walloon Excellence in Life Sciences and Biotechnology (WELBIO), Université Catholique de Louvain (UCLouvain), Brussels, Belgium

**Keywords:** *Bifidobacterium*, gut microbiota, obesity, fatty acid oxidation, liver fatty acid profile, bile acids

## Abstract

Obesity has been recognized by the World Health Organization as a global epidemic. The gut microbiota is considered as a factor involved in the regulation of numerous metabolic pathways by impacting several functions of the host. It has been suggested that probiotics can modulate host gene expression and metabolism, and thereby positively influence host adipose tissue development and obesity related-metabolic disorders. The aim of the present work was to evaluate the effect of an exopolysaccharide (EPS)-producing *Bifidobacterium* strain on host glucose and lipid metabolism and the gut microbial composition in a short-term diet-induced obesity (DIO) in mice. C57BL/6J male mice were randomly divided into three groups: a control group that received control standard diet, a group fed a high-fat diet (HF), and a group fed HF supplemented with *Bifidobacterium animalis* IPLA R1. Fasting serum insulin as well as triglycerides accumulation in the liver were significantly reduced in the group receiving *B. animalis* IPLA R1. The treatment with the EPS-producing *B. animalis* IPLA R1 tended to down-regulate the expression of host genes involved in the hepatic synthesis of fatty acids which was concomitant with an upregulation in the expression of genes related with fatty acid oxidation. *B. animalis* IPLA R1 not only promoted the increase of *Bifidobacterium* but also the levels of *Bacteroides-Prevotella*. Our data indicate that the EPS-producing *Bifidobacterium* IPLA R1 strain may have beneficial effects in metabolic disorders associated with obesity, by modulating the gut microbiota composition and promoting changes in lipids metabolism and glucose homeostasis.

## Introduction

Obesity is recognized by the World Health Organization (WHO) as a global epidemic and it results from disequilibrium between energy intake and expenditure, having a great impact in several metabolic disorders (WHO, 2017). In addition, obesity is one of the main health issues around the world due to its high prevalence ant its multifactorial etiology, which is not completely understood. The negative effect of obesity is clearly associated with an impairment of life and high health care costs. Lifestyle changes such as the increased consumption of high-energy foods have greatly contributed to the global prevalence of cardiometabolic risk factors including overweight or obesity, type 2 diabetes, but also hepatic steatosis. Therefore, there is an urgent need to identify innovative strategies to prevent or ameliorate this multifactorial disorder.

The gut microbiota is considered as an important factor partially involved in the regulation of numerous metabolic pathways by impacting different functions of the host (Duranti et al., 2017), its structure and function being strongly influenced by Western style diets. Novel approaches have been proposed to modulate the dysbiosis associated with obesity and metabolic diseases. Among them, probiotics which are live microorganism that, when administered in adequate amounts have been shown to confer health benefits to the host (Hill et al., 2014), have been extensively studied. Fermented foods, and especially dairy products, are the most popular carriers for the delivery of these microorganisms in human applications (Bigliardi and Galati, 2013). Probiotic administration can modulate host gene expression and metabolism, and thereby positively influence host adipose tissue development and obesity related-metabolic disorders (Kobyliak et al., 2016; Cerdo et al., 2019). Murine models have become essential tools for understanding the complex interactions between gut microbes, their hosts, and certain diseases. Models of diet-induced obesity (DIO), wherein animals are fed a high-fat diet (HFD) or other obesogenic diet, allow investigators to manipulate caloric intake and diet composition in genetically intact animals (Neyrinck et al., 2016). However, the observed effects and the mechanisms by which probiotics exert such effects could differ as depending on the microorganism administered and the animal model used.

Members of genus *Bifidobacterium* are commensal microorganisms found in the human gastrointestinal tract and have been traditionally considered as beneficial for human health. Some of their strains are able to produce exocellular carbohydrate polymers (EPS) with potential health benefits (Salazar et al., 2008; Castro-Bravo et al., 2018). At IPLA-CSIC, we have isolated and characterized some *Bifidobacterium* strains and their EPS using several *in vitro* models for assessing the immune response profile promoted in the host (Lopez et al., 2012) or the capacity to modulate the intestinal microbiota (Salazar et al., 2009). *In vivo* studies with two of these strains using Wistar rats have shown that these microorganisms were able to modulate the intestinal microbiota (Salazar et al., 2011). The oral administration of *B. animalis* IPLA R1 strain to healthy Wistar rats fed a standard diet did not modify the insulin-regulated trafficking of the glucose transporter GLUT4 from intracellular vesicles (endosomes) to the cell membrane of either adipose or muscular tissues and do not affect the glucose, insulin, and HOMA index in blood. Moreover, this strain reduced the pro-inflammatory IL-6 and promoted the synthesis of the regulatory cytokine TGF-β (Salazar et al., 2014). These results suggested that IPLA R1 strain could be a good candidate to be tested in low grade inflammation model as DIO model in order to evaluate the effect on metabolic disturbances (glucose and lipid homeostasis) together with inflammation.

In addition, preclinical evidence supporting the “antiobesity” effect of some probiotics has been mainly obtained using DIO mice or rats fed long-term high fat diets and supplemented with one or more different strains, mostly from *Lactobacillus* and *Bifidobacterium* genera (Bagarolli et al., 2017). However, the effects of these potential probiotics on short-term DIO animals have been considerably less explored.

The purpose of the present study was to gain insight into the effects promoted by the EPS-producing *B. animalis* IPLA R1 strain (Ruas-Madiedo et al., 2006) on the glucose and lipid metabolism and on the gut microbial community structure in short-term DIO mice.

## Materials and Methods

### Preparation of the *Bifidobacterium* Strain

Cultures of the strain *B. animalis* IPLA R1 grown overnight in MRS supplemented with 0.25% (w/v) L-cysteine (MRSC) in anaerobic conditions (anaerobic cabinet under a 10% H_2_, 10% CO_2_, and 80% N_2_ atmosphere) were used to inoculate (2% w/v) fresh MRSC broth which was incubated at 37% for 24 h. Afterwards, cultures were washed twice with sterile PBS solution and re-suspended in sterile 10%-reconstituted skimmed milk at a concentration of about 1 × 10^10^ cfu/ml and then they were freeze-dried and stored at 4°C until use. To test the viability of the strains in the milk-bacterial preparations, serial dilutions in Ringer’s solution were made from the stored freeze-dried tubes and deep plated on agar-MRSC. Plates were incubated under anaerobic conditions for 72 h to determine the bifidobacterial counts (cfu/ml).

### Animals

Nine-week-old male C57BL/6J mice were housed in groups of two mice per cage in a 12 h light/dark cycle (lights off at 6:00 pm) and were given free access to diet and water. After an acclimatization period of 1 week, mice underwent a pre-treatment period where all animals received a control diet (AIN93M; Research Diet, New Brunswick, NJ, United States). During this pretreatment period, mice were divided into 3 groups of 8 animals each: HFD administered with bifidobacteria (HF-B) were supplemented with a daily dose (∼5 × 10^8^ cfu) of *B. animalis* IPLA R1 strain suspended in 10%- skimmed milk, whereas the Control (CT) and the High fat diet (HF) groups where supplemented with a daily dose of the placebo (skimmed milk) in the drinking water. The *B. animalis* IPLA R1 dose was similar to previous studies performed in the group with Wistar rats (Salazar et al., 2011, 2014). The *Bifidobacterium* milk suspension administered in the drinking bottle per cage was changed every day and bottles specially designed to facilitate the easy access to small drinking volumes were used. The absence of a significant decrease in the viability of milk-bacterial suspensions in the drinking water maintained at 25°C during 4 days was corroborated daily by plate counting (MRSC-agar; plate incubation at 37°C in anaerobic conditions for 72 h) Ringer’s (Merck, Darmstadt, Germany) serial dilutions of the water bacterial suspensions.

After the 7 days’ pre-treatment period, the HF and HF-B groups switched to a high fat diet containing 60% lipids (soybean oil and lard), 20% protein, and 20% carbohydrates as energy content (D12492, Research Diets, New Brunswick, NJ, United States) for 3 days. Food intake, taking into account spillage, and water consumption were recorded twice a week. After 10 days, mice were anesthetized with isoflurane gas before exsanguination and tissue sampling, and the mice were then killed by cervical dislocation. Portal blood was collected, centrifuged (13000 g, 3 min) and the serum was stored at −80°C. Mice were sacrificed using cervical dislocation. The cecal content, the liver, the visceral and subcutaneous adipose tissues, were precisely dissected, collected and weighed in aseptic conditions, and they were frozen in liquid N_2_ and stored at −80°C.

### Gut Microbiota

Genomic DNA was extracted from the cecal content using a QIAamp DNA Stool Mini Kit (Qiagen, Hilden, Germany) according to the manufacturer’s instructions, including a bead-beating of 1 minute (glass beads 0,45 μm, VWR, Belgium) [Quantitative PCR (qPCR) was performed with a StepOnePlus Real-Time PCR System and software (Applied Biosystems, Den Ijssel, Netherlands] using Mesa Fast qPCR^TM^ (Eurogentec, Seraing, Belgium) for detection. The cycle threshold of each sample was compared with a standard curve made by diluting genomic DNA isolated from pure cultures of type strains (BCCM/LMG, Ghent, Belgium; DSMZ, Braunshweig, Germany). The qPCR for *Akkermansia muciniphila, Bacteroides-Prevotella, Bifidobacterium*, *B. animalis*, *Lactobacillus, Roseburia*, and total bacteria were performed as previously described (Bindels et al., 2015).

### Bile Acids

Bile acids (BA) were measured in feces using Bile Acids kit (DiaSys Diagnostic and Systems, Holzheim, Germany), following the manufacturer’s instructions.

### Tissue mRNA

Total RNA was extracted from tissues using the TriPure isolation reagent kit (Roche Diagnostics, Penzberg, Germany). Complementary DNA was prepared by reverse transcription of 1 μg total RNA using the Kit Reverse Transcription System (Promega, Madison, WI). Real-time PCR was performed with the StepOne System (Applied Biosystems, Netherlands). For adipose tissue, RNA quality was checked using an Agilent 2100 Bioanalyzer (Agilent Technologies, Santa Clara, CA, United States) with a quality threshold at 6. Samples were run in duplicate and the data were analyzed using the 2^–ΔΔCT^ method. The purity of the amplified product was verified by analyzing the melt curve performed at the end of the amplification step. The expression of the targeted gene was normalized with the expression of the ribosomal protein L19 (*Rpl19*). The primer sequences of the targeted genes are listed in Supplementary Table 1.

### Blood Biochemical Parameters

Blood glucose concentration was determined on animals before anesthesia, with a glucose meter (Roche Diagnostic, Meylan, France) on blood collected from the tip of the tail vein. Plasma insulin concentration was determined using ELISA kit (Mercodia, Upssala, Sweden). Homeostasis Model Assessment Insulin Resistance (HOMA-IR) was calculated as follows: [fasted glycemia (mM)^*^fasted insulinemia (μU/ml)]/22.5. Plasma triglycerides, cholesterol, and non-esterified fatty acids were determined by using commercial kits coupling enzymatic reaction and spectrophotometric detection of reaction in products (DyaSys Diagnostic and Systems, Holzheim, Germany). High density lipoprotein cholesterol (HDL-Cholesterol) concentration was measured enzymatically after very low density lipoprotein (VLDL), chylomicrons and low density lipoprotein cholesterol (LDL-Cholesterol) antibodies precipitation (DyaSys Diagnostic and Systems, Holzheim, Germany). Plasma concentrations of ghrelin, PYY, glucose-dependent insulinotropic polypeptide, (GIP) and glucagon-like peptide-1 (GLP-1) were quantified using a Bio-Plex Multiplex immunoassays kits (Bio-Rad, Nazareth, Belgium) and measured by using Luminex technology (Bio-Plex 200; Bio-Rad) following the manufacturer’s instructions.

### Biochemical Analyses in the Liver

Triglycerides and cholesterol were measured in the liver tissue after extraction with chloroform–methanol as previously described (Neyrinck et al., 2012). Fatty acid profile was determined in liver using gas chromatography coupled to ion flame detector as previously indicated (Druart et al., 2014b).

### Statistical Analysis

Results are presented as means with their standard error. Statistical significance of difference between groups was assessed by one-way analysis of variance (ANOVA) followed by *post hoc* Tukey’s multiple comparison tests using Graph-Pad Prism (version 6.00 for Windows, GraphPad Software, San Diego, CA, United States). Variances within-groups were compared using a Bartlett’s test. If variances were significantly different between groups, values were normalized by Log transformation before proceeding to the analysis. Data with different superscript letters are significantly different (*p* < 0.05) according to the *post hoc* Tukey ANOVA statistical analysis. Grubbs method was used to test for outliers.

## Results

### The Administration of the EPS-Producing *B. animalis* IPLA R1 Strain Does Not Significantly Affect HFD-Induced Obesity and Blood Parameters in a Short Period of Time

The short-term administration of a HFD promoted a significant increase in body weight at the end of the period of treatment in the HF group of mice accordingly to the higher energy intake (Table 1). This effect was accompanied by increased fat depots (visceral and subcutaneous adipose tissues) whereas a decrease in the caecum content was observed in the groups of mice receiving the HFD (Table 1). The administration of EPS-producing *B. animalis* IPLA R1 did not prevent the HFD-induced body weight gain and no changes in energy intake were observed with respect to the HF group not receiving the strain (Table 1). Food and water intake were not affected by the dietary treatments (data not shown).

**TABLE 1:** Energy intake and body and tissue weights.

	**CT**	**HF**	**HF-B**
Body weight gain (g)	1.02 ± 0.15^a^	2.47 ± 0.14^b^	2.07 ± 0.23^b^
Energy intake (kcal/day)	9.99 ± 0.22^a^	13.94 ± 0.61^b^	13.67 ± 0.40^b^
Cecal tissue (% body weight)	0.29 ± 0.02	0.23 ± 0.01	0.25 ± 0.01
Cecal content (g)	0.18 ± 0.01^a^	0.13 ± 0.01^b^	0.12 ± 0.01^b^
Liver (% body weight)	3.80 ± 0.09	3.95 ± 0.09	3.93 ± 0.05
*Gastrocnemius* muscle (g)	0.14 ± 0.00	0.14 ± 0.00	0.14 ± 0.00
SAT (% body weight)	1.15 ± 0.03^a^	1.47 ± 0.10^ab^	1.62 ± 0.13^b^
VAT (% body weight)	0.55 ± 0.05^a^	0.94 ± 0.03^b^	0.97 ± 0.08^b^

*Energy intake body and tissue weights after a short-term high fat diet (HFD). Mice fed a control diet and delivery vehicle-skimmed milk in drinking water (CT), mice fed a HFD and delivery vehicle-skimmed milk in drinking water (HF), and mice fed a HFD supplemented with a suspension of 5 × 10^8^ cfu/mouse/day of *B. animalis* IPLA R1 strain in skimmed milk (HF-B) added to the drinking water. CT diet (3.85 kcal/g) and HFD (5.24 Kcal/g). Data are expressed as the mean ± SEM. Data with different superscript letters are significantly different at *p* < 0.05 according to One-way analysis of variance statistical analysis followed by Tukey *post hoc*. SAT, subcutaneous adipose tissue; VAT, visceral adipose tissue.*

HFD feeding increased glycemia and cholesterolemia (total cholesterol and HDL-cholesterol). Fasting insulinemia was higher in HF group than in CT group and the administration of the *Bifidobacterium* IPLA R1 strain counteracted the HFD effect. The significant decrease of insulin was not associated with changes in fasted glycemia, which remain higher in both the HFD and the *Bifidobacterium* IPLA R1 strain treated mice. In accordance with this result, the HFD promoted an increase of the insulin resistance index (HOMA) value and the administration of the *Bifidobacterium* IPLA R1 strain tended to counteract this effect although did not reach statistical significance with HF group (Table 2).

**TABLE 2:** Serum parameters.

	**CT**	**HF**	**HF-B**
Non-esterified fatty	0.39 ± 0.03	0.36 ± 0.03	0.41 ± 0.05
acids (mM)			
Triglycerides (mM)	0.48 ± 0.01	0.56 ± 0.04	0.46 ± 0.03
Glycemia (mg/dl)	134.50 ± 5.21^a^	176.90 ± 3.13^b^	177.30 ± 5.54^b^
Insulinemia (pM)	35.02 ± 12.94^a^	71.29 ± 11.83^b^	36.98 ± 6.72^a^
HOMA	1.74 ± 0.67^a^	4.52 ± 0.79^b^	2.59 ± 0.43^ab^
Cholesterol (mM)	1.50 ± 0.05^a^	1.98 ± 0.07^b^	1.97 ± 0.06^b^
HDL-CHO (mM)	0.08 ± 0.00^a^	0.12 ± 0.00^b^	0.13 ± 0.01^b^
Ghrelin (pg/ml)	756.10 ± 165.00	426.80 ± 66.93	453.30 ± 69.65
PYY (pg/ml)	39.23 ± 10.60	60.79 ± 2.50	54.85 ± 6.38
GIP (pg/ml)	62.47 ± 8.79	76.18 ± 8.47	62.42 ± 7.84
GLP-1 (pg/ml)	208.70 ± 40.42	273.40 ± 64.98	187.70 ± 67.83

*Serum parameters after a short-term high fat diet (HFD). Mice fed a control diet and delivery vehicle-skimmed milk in drinking water (CT), mice fed a HFD and delivery vehicle-skimmed milk in drinking water (HF), and mice fed a HFD supplemented with a suspension of 5 × 10^8^ cfu/mouse/day of *B. animalis* IPLA R1 strain in skimmed milk (HF-B) added to the drinking water. Data are expressed as the mean ± SEM. Data with different superscript letters are significantly different at *p* < 0.05 according to One-way analysis of variance statistical analysis followed by Tukey *post hoc*. GIP, glucose-dependent insulinotropic polypeptide; GLP-1 glucagon like peptide-1; HDL-CHO, high density lipoprotein cholesterol (HDL-Cholesterol). HOMA, homeostasis model assessment.*

The plasma levels of non-esterified fatty acids, triglycerides, ghrelin, PYY, GIP and GLP-1 were not significantly affected by the short period HFD feeding or the supplementation with the *Bifidobacterium* IPLA R1 strain (Table 2).

### EPS-Producing *B. animalis* IPLA R1 Administration Does Not Affect Inflammatory Status of the Gut and the Adipose Tissue

Obesity is generally associated with a low-grade inflammation in the gut but also in the peripheral tissues, principally in adipose tissue. We measured the mRNA expression of different inflammatory markers in the jejunum, ileum and the colon (Supplementary Table 2). In the ileum, we observed that HFD feeding significantly increased tumor necrosis factor (*Tnf)* and C-C motif chemokine ligand 2 (*Ccl2)* mRNA expression, whereas the supplementation of the EPS-producing *B. animalis* IPLA R1 did not counteract the effect of the HFD (Supplementary Table 2). Additional markers were analyzed in the ileum to evaluate the gut barrier integrity, such as the expression of tight junction proteins [Occludin (OCLN) and tight junction protein 1 (TJP1)] or mucin 2 (MUC-2) but none of them was affected by the treatments (Supplementary Table 2). Furthermore, we did not observe any statistically significant difference for the pro-inflammatory markers tested in the subcutaneous adipose tissue (SAT) or in the visceral adipose tissue (VAT) whatever the dietary treatment (Supplementary Table 2).

### EPS-Producing *B. animalis* IPLA R1 Administration Promoted Changes in Liver Lipid Content and Glucose Metabolism Markers

**FIGURE 1:**
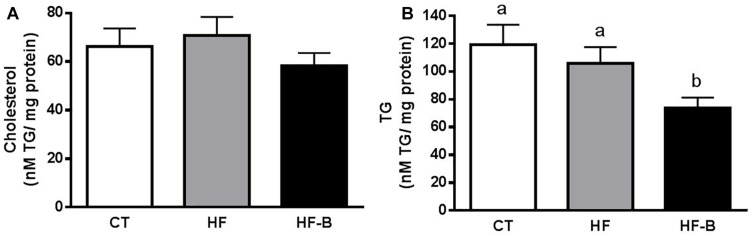
Impact of the EPS-producing *B. animalis* IPLA R1 strain after a short-term high fat diet (HFD) on lipid accumulation in the liver. Liver cholesterol **(A)** and triglycerides (TG) **(B)**. Mice fed a control diet and delivery vehicle-skimmed milk in drinking water (CT), mice fed a HFD and delivery vehicle-skimmed milk in drinking water (HF), and mice fed a HFD supplemented with a suspension of 5 × 10^8^ cfu/mouse/day of *B. animalis* IPLA R1 strain in skimmed milk (HF-B) added to the drinking water. Data are expressed as the mean ± SEM. Data with different superscript letters are significantly different at *p* < 0.05 according to One-way analysis of variance statistical analysis followed by Tukey *post hoc*.

The administration of HFD during 3 days supplemented with the strain *B. animalis* IPLA R1 promoted a decrease of the hepatic lipids content (Figures 1A,B), which reached statistical significance for triglycerides (Figure 1B).

**FIGURE 2:**
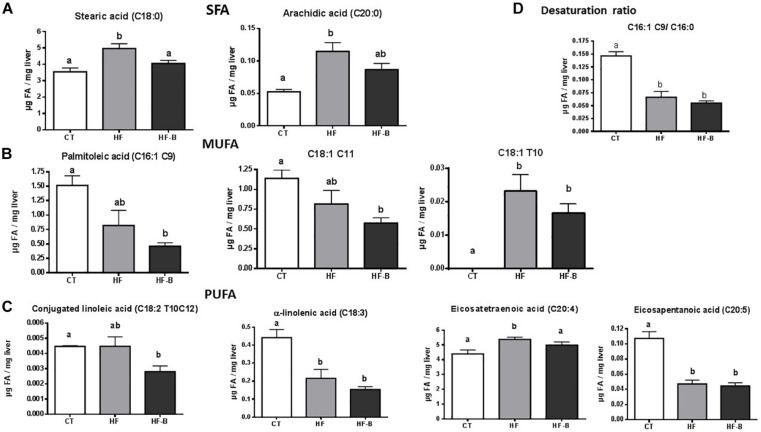
Statistically significant changes on liver fatty acid profile after a short-term high fat diet (HFD). Saturated fatty acids (SFA) profile. **(A)** Liver monounsaturated fatty acids (MUFA) profile **(B)**. Liver polyunsaturated fatty acids (PUFA) profile **(C)**. Desaturation ratio **(D)**. Mice fed a control diet and delivery vehicle-skimmed milk in drinking water (CT), mice fed a HFD and delivery vehicle-skimmed milk in drinking water (HF), and mice fed a HFD supplemented with a suspension of 5 × 10^8^ cfu/mouse/day of *B. animalis* IPLA R1 strain in skimmed milk (HF-B) added to the drinking water. Data are expressed as the mean ± SEM. Data with different superscript letters are significantly different at *p* < 0.05 according to One-way analysis of variance statistical analysis followed by Tukey *post hoc*.

**FIGURE 3:**
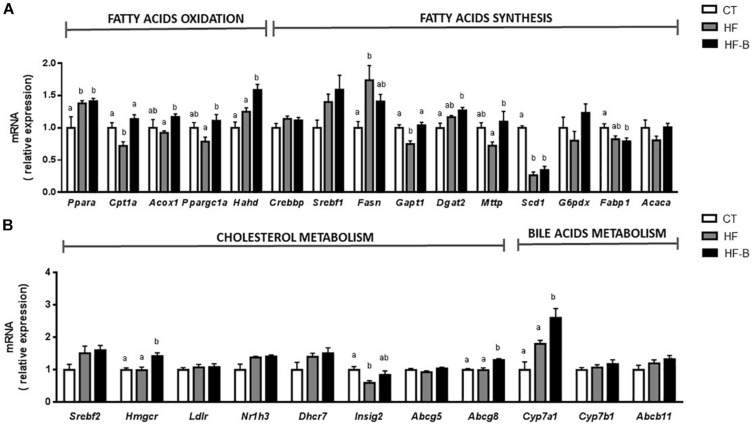
Liver gene expression of fatty acids metabolism **(A)** and gene expression of cholesterol and bile acid metabolism **(B)** after a short-term high fat diet (HFD). Mice fed a control diet and delivery vehicle-skimmed milk in drinking water (CT), mice fed a HFD and skimmed milk in drinking water (HF), and mice fed a HFD and supplemented with a suspension of 5 × 10^8^ cfu/mouse/day of *B. animalis* IPLA R1 strain in skimmed milk (HF-B) added to the drinking water. Each gene codes for the same name of the corresponding enzyme except *Acaca* that codes for ACC (Acetyl-CoA carboxylase) and *Nr1h3* that codes for LXR (liver X receptor). Data are expressed as the mean ± SEM. Values are expressed as relative units with the mean of CT mice values set at 1. Data with different superscript letters are significantly different at *p* < 0.05 according to One-way analysis of variance statistical analysis followed by Tukey *post hoc*.

Fatty acid composition of the liver after a short period of HFD administration evidenced variations with respect to animals fed standard diet (Figure 2 and Supplementary Table 3). Among changes induced by the HFD, it is worth mentioning a significant increase of stearic (C18:0) and arachidic (C20:0) acids compared to CT mice; remarkably, the supplementation with the EPS-producing strain IPLA R1 blunted the effect of the HFD on these two fatty acids (Figure 2A). Shifts were also found for monounsaturated fatty acids (MUFA) such as palmitoleic acid [C16:1 (cis-9)] and C18:1 (cis-11), that decreased in animals fed HFD and reached statistically significance only when bifidobacteria were administrated concomitantly (Figure 2B) whereas C18:1 (trans 10) increased significantly in both HFD fed groups. Among PUFA, conjugated linoleic acid [C18:2 (trans 10, cis 12)] significantly decreased in the HF-B group as compared to CT group and eicosatetraenoic acid (C20:4) significantly decreased in HF-B group as compared to HF group whereas α-linoleic acid (ALA, C18:3) and eicosapentaenoic acid [EPA, C20:5 (n-6)] decreased significantly in both HFD fed groups of mice (Figure 2C). In addition, HFD feeding caused a decrease on the C16:1 C9/C16:0 desaturation ratio independently of the bifidobacteria administration (Figure 2D). Coherently, the expression of stearoyl-CoA desaturase (*Scd1*) was downregulated in the liver by feeding HFD (Figure 3A). No significant differences were found among the three groups of mice for C18:1 C9/C16:0 desaturation ratio (Supplementary Table 3).

Based on the decrease of hepatic triglycerides in the group of mice that received the HFD supplemented with the EPS-producing *Bifidobacterium* strain, we measured the levels of mRNA coding for key enzymes involved in hepatic β-oxidation such as peroxisome proliferator-activated receptor α (PPARA) and their regulated gene products: carnitine palmitoyl transferase 1A (CPT1A), acyl-CoA oxidase 1 (ACOX1), PPARG coactivator 1 alpha (PPARGC1A), and hydroxyacyl-CoA dehydrogenase (HADH). Interestingly, the levels of *Acox1*, *Cpt1a*, *Ppargc1a* and *Hahd* genes in the liver were significantly higher in mice fed with EPS-producing *B. animalis* R1 strain as compared to the HF group of mice not receiving the bifidobacteria. On the other hand, the mRNA expression coding for enzymes involved in fatty acid synthesis such as sterol regulatory element transcription factor 1 (SREBF1) and its targeted genes, fatty acid synthase (*Fasn*) and acetyl CoA carboxylase (*Acaca*), were determined. Only *Fasn* was significantly upregulated upon HFD feeding and a moderate decrease in the expression of this enzyme was observed in the HF-B group although did not reach statistical significance (Figure 3A). Interestingly, the administration of *B. animalis* R1 strain promoted an upregulation of the expression of glycerol-3-phosphate acyltransferase (*Gpat1*), a key enzyme involved in triacylglycerol synthesis.

Strikingly, the administration of the strain IPLA R1 in mice fed HFD, increased significantly the mRNA expression of 3-hydroxy-3-methyl-3-glutaryl-CoA reductase (*Hmgcr*) that codifies for a limiting enzyme in the process of hepatic cholesterol synthesis (Figure 3B). The HF-B group also displayed increased levels of the *Cyp7a1*, which is involved in the classical route to bile acid synthesis, and of *Abcg8* gene that is involved in the transport of cholesterol. The mRNA expression of the gene coding for the microsomal triglycerides transfer protein (MTTP), involved in the transport of triglycerides and which is essential for VLDL synthesis, was also significantly higher in the group of mice that received the HFD supplemented with the bifidobacteria as compared to the HF group. Although significant higher levels of fecal bile acids were found in HF compared to CT group, no any differential effect for the *Bifidobacterium* IPLA R1 strain was evidenced (3.99 ± 0.20, 6.06 ± 0.07 and 6.36 ± 0.28 μM for CT, HF and HF-B groups, respectively, *p* > 0.05, ANOVA).

### Effect of the EPS-Producing Strain *B. animalis* IPLA R1 on the Gut Microbial Community

**FIGURE 4:**
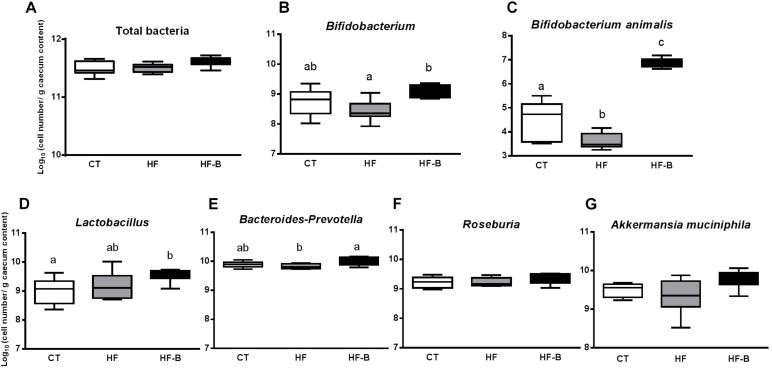
Impact of the EPS-producing *B. animalis* IPLA R1 strain after a short-term high fat diet (HFD) on cecal microbiota assessed by qPCR. Total bacteria **(A)**, *Bifidobacterium* **(B)**, *B. animalis* **(C)**, *Lactobacillus* **(D)**, *Bacteroides-Prevotella* **(E)**, *Roseburia* **(F)**, and *A. muciniphila* **(G)** in the cecal content of mice after 3 days of treatment. Mice fed a control diet and delivery vehicle-skimmed milk in drinking water (CT), mice fed a HFD and delivery vehicle-skimmed milk in drinking water (HF), and mice fed with HFD supplemented with a bacterial suspension in milk (5 × 10^8^ cfu/mouse/day) of *B. animalis* IPLA R1 strain (HF-B) added to the drinking water. Data are box and whiskers plots with minimum and maximum. Data with different superscript letters are significantly different at *p* < 0.05 according to One-way analysis of variance statistical analysis followed by Tukey *post hoc.*

Some intestinal bacteria are known to be involved in the regulation of gut barrier function and/or inflammatory processes. *Bifidobacterium*, *B. animalis*, *Lactobacillus*, *Bacteroides-Prevotella*, *Roseburia*, and *A. muciniphila* were analyzed by qPCR in the cecal content of our mice groups receiving different diets (Figure 4). The levels of fecal *Bifidobacterium* in mice were measured before the pre-treatment period and after the study period. The initial number of fecal bifidobacteria were not significantly different in all groups (9.20 ± 0.23, 8.92 ± 0.16 and 9.32 ± 0.15 log_10_ cell number/g feces for CT, HF and HF-B groups, respectively, *p* > 0.05, ANOVA) whereas fecal *Bifidobacterium* levels after the study period were, as expected, significantly higher in the HF-B group vs. HF group (9.70 ± 0.28^ab^, 9.02 ± 0.14^a^ and 10.16^b^. ± 0.14 log_10_ cell number/g feces for CT, HF and HF-B groups, respectively, *p* < 0.05, ANOVA).

At the end of the experimental period, the cecal content of bifidobacteria, in particular *B. animalis*, increased in HF-B mice group compared to the HF or CT groups and *Lactobacillus* increased significantly in HF-B group compared to CT group (Figures 4B,D). There was also a reduction in *Bacteroides-Prevotella* levels in the HFD fed group compared to CT group that was restored by the EPS-producing *B. animalis* IPLA R1 administration (Figure 4). There were no differences between the three mice groups for total bacteria, *Roseburia* and *A. muciniphila* levels whatever the experimental group considered is (Figure 4).

## Discussion

There is an increasing interest on understanding the influence of probiotics in human health. Preclinical evidence supporting their capacity to alleviate obesity have been mainly carried out in DIO rodents whose diets were supplemented with a variety of *Lactobacillus* and *Bifidobacterium* strains, either individually or in combination (Druart et al., 2014a; Kobyliak et al., 2016; Dahiya et al., 2017). However, the anti-obesity effect of probiotics seems to be strain and dose dependent and the underlying mechanisms of their action remain largely unknown. *In vivo* trials, the decrease of body weight and fat mass and other features associated with obesity such as insulin resistance, low-grade inflammation, steatosis, fatty acids metabolism and gut microbiota changes have been studied. Moreover, the probiotic supplementation has traditionally been performed for relatively long periods of time (between 3 and 18 weeks) (Neyrinck et al., 2016; Bagarolli et al., 2017; Karimi et al., 2017; Ray et al., 2018). Concerning *B. animalis* species, some recent studies performed in mice fed with a HFD for a long-term period of time have evidenced that the administration of some strains of this species contributed to reduce body fat content, attenuated glucose intolerance and ameliorated metabolic endotoxemia, and adipose and hepatic inflammation (Chen et al., 2012; Stenman et al., 2014; Caimari et al., 2017). In the present study, we found no changes in food and water intake between the three experimental groups of animals, and the supplementation with *B. animalis* IPLA R1 did not prevent body weight gain, probably due to the short term period of dietary intervention. Previous studies have reported that a short treatment (few days) with a HFD increased body weight (Lanthier et al., 2010; Lee et al., 2011) whereas other authors did not found any effect on weight gain (Chiazza et al., 2016). Probably, differences in dietary composition, housing conditions, genetic background or age at dietary intervention may have contributed to the contradictory results obtained in body weight gain by different authors. A previous study on DIO mice supplemented with *Bifidobacterium* strains during long periods of time did not show any influence in body weight gain of the microorganism administered (Chen et al., 2012).

In our experimental groups of mice, fasted glycemia and insulinemia were significantly higher due to HFD feeding, suggesting that the short period of HFD treatment (3 days) was enough to promote changes in glucose homeostasis, these results suggest that the model of short-term HFD is useful to reproduce some of the classical features associated with obesity, such as an altered glucose metabolism. This model has been employed previously by other authors in rodents in which they observed that HFD feeding for short periods ranging between 3 and 7 days were enough to produce an impairment of glucose tolerance and hepatic insulin sensitivity by different mechanisms including Kuppfer cell activation (Lanthier et al., 2010) and adipose tissue inflammation (Wiedemann et al., 2013) and favored the development of steatosis (Lanthier et al., 2010; Lee et al., 2011; Wiedemann et al., 2013; Chiazza et al., 2016). Interestingly, the administration in our study of *B. animalis* IPLA R1 strain promoted a decrease of serum insulin levels, not accompanied by a significant variation in fasting glucose and HOMA index, suggesting a slight improvement of insulin sensitivity in animals fed HFD supplemented with the *B. animalis* IPLA R1 strain. Moreover, the HF-B group showed lower hepatic triglyceride levels as compared to the HF group. Previous reports have correlated the positive effect of the genus *Bifidobacterium* with improved glucose tolerance and glucose-induced insulin secretion in HFD and prebiotic-treated mice (Cani et al., 2007; Blaut and Bischoff, 2010). In addition, previous studies evaluating the effect of the administration of different *Bifidobacterium* strains in obese mice have also shown a decrease in insulin resistance (Chen et al., 2012; Cano et al., 2013). The results from the present work demonstrate for the first time that only 3 days HFD supplemented with a *B. animalis* IPLA R1 strain was enough to reduce fasted insulinemia. As a perspective, it could be interesting to check for further improvements upon a long term HFD with IPLA R1 strain administration.

DIO models are classically associated with low grade inflammation in different tissues (Delzenne et al., 2011). The very short time HFD feeding used in our *in vivo* study was not sufficient to induce inflammation in the adipose tissues but it induced inflammation in the gut (ileum). The gut inflammation in the group of mice fed HFD is in accordance with a previous work performed in rodent models where the HFD consumption leads to intestinal inflammation associated with microbial dysbiosis (Porras et al., 2017).

It is well known that dietary fats, probiotic and prebiotic administration can modulate the fatty acid composition of various organs in the host (Wall et al., 2010; Druart et al., 2013; Patterson et al., 2017). Only one study is currently available investigating the influence of the administration of two different *Lactobacillus* strains on the host’s liver fatty acid profile composition during a period of 16 weeks of HFD feeding (Ivanovic et al., 2016). Here, we investigated the impact of HFD and the supplementation with the *Bifidobacterium* IPLA R1 strain administered during a very short period of time on the liver fatty acid profile. Higher content of SFA [arachidic (C20:0) and stearic (C18:0) acids] and one PUFA [eicosatetrenoic acid (C20:4)] were observed in the liver of mice receiving HFD during 3 days, which is in agreement with previous studies performed in mice (Druart et al., 2013; da Silva-Santi et al., 2016). The higher content of SFA in the liver of HFD group could be related with the diet composition as lard and soybean oil were the major source of dietary fat in HFD which contained 85.5 g of SFA in contrast to the 5.7 g in the control diet, according to composition provided by the manufacturer. The administration of the *Bifidobacterium* IPLA R1 strain caused a significant decrease of the stearic and eicosatetraenoic fatty acids levels as compared to the administration of the HFD alone. These results are in accordance with a previous study showing that the administration of the strain *Lactobacillus rhamnosus* LA68 in HFD feeding for 12 weeks caused significant changes in certain SFA. The same authors have obtained different effects when mice under HFD were suplemented with *Lactobacillus plantarum* WCFS1, finding an increase in SFA and PUFA n-6 series (Ivanovic et al., 2016). This variability on results suggest that the different pattern observed in liver fatty acid profile is strain dependent. In adition, the HFD administration caused a decrease on the mRNA expression of *Scd1* and SCD1 activity measured in the present work by the study of two different desaturation ratios (C16:1 C9/C16:0 and C18:1 C9/C16:0). SCD1 is the rate-limiting enzyme catalyzing the conversion of saturated long chain fatty acids into MUFA which are major components of triglycerides. The preferred substrates are palmitoyl-CoA (16:0) and stearoyl-CoA (18:0), which are converted into palmitoleoyl-CoA (16:1), and oleoyl-CoA (18:1), respectively. The resulting MUFA are major components of triglycerides, cholesterol esters, and phospholipids.

Our results are in agreement with previous studies carried out in our group with mice fed with HFD where we have also observed a decrease in the expression and activity of SCD1 after HFD feeding (Druart et al., 2013). It has also been reported that the activity of SCD1 is inhibited by HFD containing high PUFA levels (Vessby et al., 2002; Ntambi and Miyazaki, 2004; da Silva-Santi et al., 2016). In this regard, the HFD employed in the present work according to the manufacturer, contained 81.5 g of PUFA with respect to the 24.5 g in the control diet. Nevertheless, the administration of the *Bifidobacterium* IPLA R1 strain to mice fed HFD did not promote any modification on the desaturation ratios. Since an increase in fatty acid oxidation is suggested by the analysis of the gene expression in the liver of animals supplemented with *B. animalis* IPLA R1, this event could be considered to explain the decrease in SFA- which are largely prone to be oxidized as compared to PUFA- in the liver tissue when HFD fed mice received the *Bifidobacterium* IPLA R1 strain.

The liver is one of the most important metabolic organs in the body and the main site of the “novo lipogenesis.” To elucidate the potential mechanisms of EPS-producing strain *B. animalis* IPLA R1 involved in the reduction of hepatic triglycerides, we screened the expression of liver genes related to lipid metabolism. PPARA is a master regulator of the expression of genes involved in lipid metabolism, by controlling the expression of genes involved in mitochondrial and peroxisomal β-oxidation (Ahmed et al., 2007). The present study revealed that the administration of *B. animalis* IPLA R1 significantly increased mRNA expression of *Acox1*, *Cpt1a, Ppargc1a* and *Hahd* compared to the HF group. ACOX1 and CPT1 are rate limiting enzymes in mitochondrial fatty acid oxidation of saturated and unsaturated fatty acids (Ouali et al., 2000). CPT1A is essential for the transport of long chain fatty acids into the mitochondria and HAHD is involved in the second step of β-oxidation of saturated and unsaturated fatty acids. Regarding fatty acid synthesis, SREBF1 is considered as a transcription factor that regulates the expression of downstream target genes involved in glucose utilization and fatty acid synthesis, such as *Fasn* and *Scd1* (Ferre and Foufelle, 2007). Although no significant differences were found between the three experimental mice groups for *Srebpf1*, it was observed a significant increase of *Fasn*, the limiting enzyme of fatty acid synthesis in the liver, in HFD group as well as a decrease in the expression of the *Scd1* in HF and HF-B groups. It has been reported that decreased expression in the *Scd1* gene inhibits lipogenesis and facilitates fatty acid oxidation, and thus suppresses triglyceride accumulation (Am et al., 2017). All these results suggest that the higher rate of triglyceride secretion through the higher expression of *Mttp* together with the upregulation of fatty acid oxidation pathway could partly account for the lower levels of triglycerides in the liver found in the group of mice that received the HFD supplemented with the bifidobacteria. Moreover, the upregulation of β-oxidation by *B. animalis* IPLA R1 can potentially lead to improved insulin sensitivity, since fatty acid derivatives influence insulin signaling in the liver tissue (Delzenne et al., 2015). We cannot exclude that the beta-oxidation process can be influenced indirectly through the production of short chain fatty acids (SCFA) by the gut microbiota because SCFA were recently shown to increase intestinal gluconeogenesis, resulting in beneficial effects on insulin sensitivity (De Vadder et al., 2014). It has been also proposed that SCFA downregulate peroxisome proliferator-activated receptor-γ (PPARγ) via AMPK promoting fat oxidation in liver and adipose tissue and improving insulin sensitivity (den Besten et al., 2015) although these hypotheses deserve future experimentation. In this respect, a previous study from our research group with healthy adult Wistar rats administered *B. animalis* IPLA R1, evidenced significant changes in the fecal and cecal profile of SCFA upon intervention (Salazar et al., 2011). Future research could be elaborated to assess the relevance of SCFA as mediators of the IPLA R1 strain effects.

The liver is also the main organ for whole body cholesterol homeostasis. We have measured the expression of genes involved in cholesterol and bile acid metabolism in the liver. The administration of the *Bifidobacterium* IPLA R1 strain promoted increased expression of *Hmgcr* gene, a limiting enzyme in the process of hepatic cholesterol synthesis in the group of mice supplemented with the strain IPLA R1 compared to CT and HF groups. Moreover, an increase in bile acids synthesis and excretion seems also to occur since *Cyp7a1*, which is involved in the classical route of bile acid synthesis and *Abcg8*, that is involved in the transport of cholesterol, were upregulated in the group receiving the probiotic strain (Chiang, 2009). The above results indicate that *B. animalis* IPLA R1 supplementation can help to prevent hepatic fat accumulation through the modulation of lipid oxidation and cholesterol excretion. Mechanisms of action of probiotics against obesity are still not clear (Kumar et al., 2012; Reis et al., 2017). Surface components of probiotic envelopes are claimed to be the molecules that establish an initial interaction, either with eukaryotic receptors or with other members of the intestinal microbiota. In this scenario, EPS produced by members of the gut microbiota, or by probiotic microorganisms ingested with foods, can be active players (Castro-Bravo et al., 2018).

One of the beneficial effects attributed to certain EPS-producer strains is the capacity to reduce cholesterol although the exact mechanisms are not well known. It has been proposed that bacterial EPS can act as some dietary fiber that, through their binding properties, can favor the elimination of bile acids in feces. EPS can also act as fermentable substrates increasing the number of gut microorganisms that can deconjugate bile acids. The subsequent decrease in bile acid reabsorption could result in the synthesis of new bile acids from cholesterol by the liver, thereby decreasing the level of circulating cholesterol (Nampoothiri et al., 2017). However, in the present study the excretion of fecal bile acids increased in mice fed HFD and the administration of the EPS-producing bifidobacteria IPLA R1 strain did not exert any additional effect. The metabolic effect of the EPS-producing bifidobacteria strain could be mediated through the modulation of bile acid metabolism since bifidobacteria are well recognized to exhibit bile salt hydrolase activities (Jarocki et al., 2014). The hypothesis of a role of bile acids would require a dynamic analysis of the enterohepatic cycle of bile acids upon *Bifidobacterium* administration. Moreover, to decipher the relevance of EPS production by this strain and its potential biological properties, it would have been desirable to evaluate separately the effect of the isolated EPS and the potential effect of the vehicle (skimmed milk). However, this experimental design is not currently affordable because of the very low amount of heteropolysaccharide isolated from *Bifidobacterium* cultures and by the difficulty to obtain polymers of high purity in laboratory conditions (Salazar et al., 2008).

The supplementation of HFD with *B. animalis* IPLA R1 modified gut microbiota and promoted an increase not only of the genus *Bifidobacterium* and the species *B. animalis* specifically in HF-B group, but also modified other bacterial populations, as *Bacteroides-Prevotella* which increased in mice supplemented with the *Bifidobacterium* IPLA R1 strain. These results confirm that the supplementation with selected probiotics during short-term HFD administration to mice is enough to promote changes in the composition of the gut microbiota and confirmed previous studies where HFD feeding significantly changed gut microbiota composition, in particular promoting a decrease in bifidobacteria (Turnbaugh et al., 2008; Cani and Everard, 2016; Neyrinck et al., 2016). The data from the present rodent model are also in line with previous work where an increase of the intestinal Firmicutes/Bacteroidetes ratio in obese leptin deficient *ob/ob* mice (Ley et al., 2005), in wild-type animals receiving Western diets (Turnbaugh et al., 2008) and in obese human (Ley et al., 2006) have been reported although in human studies these results remain controversial (Duncan et al., 2008; Schwiertz et al., 2010). Moreover, it has previously also been reported that cohousing lean and obese mice prevented the development of increased adiposity which is associated with the transfer of certain *Bacteroides* species (Ridaura et al., 2013) and the oral administration of *Bacteroides uniformis* CET 7771 or *Bacteroides thetaiotamicron* VPI-5482 strains reduced metabolic disorders and immunological dysfunction in HFD induced obese mice and protected mice against adiposity, respectively (Gauffin Cano et al., 2012; Liu et al., 2017).

In conclusion, the administration of the EPS producing *B. animalis* IPLA R1 strain has a protective effect against short-term DIO metabolic effects in mice by (1) promoting hepatic fatty-acid oxidation and decreasing fatty acid synthesis, thereby leading to a lower fat accumulation in the liver, (2) decreasing insulinemia, and (3) improving glucose metabolism. The exact mechanisms that link the administration of *B. animalis* IPLA R1 in the regulation of gut inflammation and lipid metabolism remain to be better elucidated but shifts in the gut microbiota could be evoked in the mechanism of action of PLA R1 strain in obesity-related metabolic disorders. Moreover, the relevance of EPS production must be unraveled in order to know if the development of such strains could be interesting in the management of obesity-related diseases.

## Data Availability

The datasets generated for this study are available on request to the corresponding author.

## Ethics Statement

All the mouse experiments were approved by and performed in accordance with the guidelines of the local ethics committee for animal care of the Health Sector of the Université catholique de Louvain under the supervision of Prof. F. Lemaigre and Prof. J. P. Dehoux and under the specific agreement numbers 2014/UCL/MD/022. Housing conditions were as specified by the Belgian Law of May 29, 2013, on the protection of laboratory animals (Agreement LA 1230314). Every effort was made to minimize animal pain, suffering, and distress and to reduce the number of animals used.

## Author Contributions

NS, AN, and ND conceived and designed the experiments, and drafted the manuscript. NS, AN, LB, and CD conducted the research. NS, AN, and CD analyzed the data or performed the statistical analysis. AN, PC, LB, PR-M, PC, and CR-G provided the intellectual input on the manuscript. ND planned and supervised all the experiments. All authors critically reviewed the manuscript and approved the final version to be submitted for publication.

## Conflict of Interest Statement

The authors declare that the research was conducted in the absence of any commercial or financial relationships that could be construed as a potential conflict of interest.
