# Supplementary material for: Functional Effects of EPS-Producing Bifidobacterium Administration on Energy Metabolic Alterations of Diet-Induced Obese Mice
Source: Front Microbiol. 2019 Aug 7;10:1809. doi: 10.3389/fmicb.2019.01809 (PMC6693475; doi:10.3389/fmicb.2019.01809)
Supplement: Supplementary file 1 [file Table_1.docx]

**Supplementary Table 1: Quantitative Real-time PCR primers for the targeted mouse genes**

| ***Gene symbol (Alias)*** | **Gene name** | **Forward sequence (5’-3’)** | **Reverse sequence (5’-3’)** |
| --- | --- | --- | --- |
| *Rpl19* | Ribosomal protein L19 | GAAGGTCAAAGGGAATGTGTTCA | CCTTGTCTGCCTTCAGCTTGT |
| *Crebbp*  *Pppara* | CREB binding protein  Peroxisome proliferator activated receptor alpha | GTCCGATATCTCCGACACACTCTT  CAACGGCGTCGAAGACAAA | CATTGCCCACATAAGCGTCTTCTG  TGACGGTCTCCACGGACAT |
| *Cpt1a* | Carnitine palmitoyltransferase 1 | AGACCGTGAGGAACTCAAACCTAT | TGAAGAGTCGCTCCCACT |
| *Acox1* | Acyl-CoA oxidase 1 | CTATGGGATCAGCCAGAAAGG | AGTCAAAGGCATCCACCAAAG |
| *Ppargc1a* | PPARG coactivator 1 alpha | AGCCGTGACCACTGACAACGAG | GCTGCATGGTTCTGAGTGCTAAG |
| *Hahd* | Hydroxyacyl-CoA dehydrogenase | GGGTCCGTTTGAGCTTCTT | TCTTCTTGCCCAGCTTCTTC |
| *Srebf1* | Sterol regulatory element biding transcription factor 1 | GATCAAAGAGGAGCCAGTGC | TAGATGGTGGCTGCTGAGTG |
| *Fasn* | Fatty acid synthase | TTCCAAGACGAAAATGATGC | AATTGTGGGATCAGGAGAGC |
| *Gpat1* | 1-acylglycerol-3-phosphate O-acyltransferase 1 | GTCCTGCGCTATCATGTCCA | GGATTCCCTGCCTGTGTCTG |
| *Dgat2* | Diacylglycerol O-acyltransferase 2 | ACTCTGGAGGTTGGCACCAT | GGGTGTGGCTCAGGAGGAT |
| *Scd1* | Stearoyl-CoA desaturase | CCTCTTCGGGATTTTCTACTACATG | GCCGTGCCTTGTAAGTTCTGT |
| *Mttp* | Microsomal trygliceride transfer protein | ATGATCCTCTTGGCAGTGCTT | TGAGAGGCCAGTTGTGTGAC |
| *G6pd* | Glucose-6-phosphate dehydrogenase | AGGAAGGATGGAGGAAGGAA | TGGAACCAGATGGGAAAGAG |
| *Fabp1* | Fatty acid binding protein 1 | GCAGAGCCAGGAGAACTTTG | TGATGTCCTTCCCTTTCTGG |
| *Acaca (*ACC*)* | Acetil-CoA carboxilase alpha | GTTGAGACGCTGGTTTGTAGAA | GGTCCTTATTATTGTCCCAGACGTA |
| *Srebf2* | Sterol regulatory element binding transcription factor 2 | GTGCGCTCTCGTTTTACTGAAGT | GTATAGAAGACGGCCTTCACCAA |
| *Hmgcr* | 3-hydroxil-3-methylglutarayl-CoA reductase | CCTGACACTGAACTGAAGCG | TCTTTCCAGAACACAGCACG |
| *Nr1h3* (LXR) | Nuclear receptor subfamily 1 group H member 3 | CAAGGGAGCACGCTATGTCT | CTTGCCGCTTCAGTTTCTTC |
| *Cyp7a1* | Cytochrome P450 family 1 subfamily A member 1 | GGGATTGCTGTGGTAGTGAGC | GGTATGGAATCAACCCGTTGTC |
| *Cyp7b1* | Cytochrome P450 family 1 subfamily B member 1 | TAGGCATGACGATCCTGAAA | TCTCTGGTGAAGTGGACTGAAA |
| *Ldlr* | Low density lipoprotein receptor | CTGTGGGCTCCATAGGCTATCT | GCGGTCCAGGGTCATCTTC |
| *Abcg8* | ATP binding cassette subfamily G member 8 | CCGTCGTCAGATTTCCAATGA | GGCTTCCGACCCATGAATG |
| *Abcg5* | ATP binding cassette subfamily G member 5 | TGGCCCTGCTCAGCATCT | ATTTTTAAAGGAATGGGCATCTCTT |
| *Abcb11* | ATP binding cassette subfamily B member 11 | AGATACAACCGAAGGGGACA | TCAACTTCTTCCACAAGCACA |
| *Dhcr7* | 7-dehydrocholesterol reductase | TCCAGGTGCTGCTTTATTCC | GCAGCCCATTCACCTCATAC |
| *Insig2* | Insulin induced gene 2 | TGTGAGCTGGACTAGCTTGCT | CCTAAGCCGTAAAACAAAATG |
| *Ccl2* (MCP1) | C-C motif chemokine ligand 2 | GCAGTTAACGCCCCACTCA | CCCAGCCTACTCATTGGGATCA |
| *Itgax* (CD11c) | Integrin alpha X | ACGTCAGTACAAGGAGATGTTGGA | ATCCTATTGCAGAATGCTTCTTTACC |
| *Adgre1* (F4/80) | Adhesion G protein-coupled receptor E1 | TGACAACCAGACGGCTTGTG | GCAGGCGAGGAAAAGATAGTGT |
| *Cd68* | CD68 molecule | CTTCCCACAGGCAGCACAG | AATGATGAGAGGCAGCAAGAGG |
| *Il6* | Interleukin 6 | ACAAGTCGGAGGCTTAATTACACAT | TTGCCATTGCACAACTCTTTTC |
| *Il1b* | Interleukin 1β | TCGCTCAGGGTCACAAGAAA | CATCAGAGGCAAGGAGGAAAAC |
| *Tnf* (TNF-α) | Tumor necrosis factor | AGCCCCCAGTCTGTATCCTT | GGTCACTGTCCCAGCATCTT |
| *Reg3g* | Regenerating family member 3 gamma | TTCCTGTCCTCCATGATCAAA | CATCCACCTCTGTTGGGTTC |
| *Tjp1* (ZO-1) | Tight junction protein 1 | TTTTTGACAGGGGGAGTGG | TGCTGCAGAGGTCAAAGTTCAAG |
| *Ocln* | Occludin | ATGTCCGGCCGATGCTCTC | TTTGGCTGCTCTTGGGTCTGTAT |
| *Muc2* | Mucin 2 | ATGCCCACCTCCTCAAAGAC | GTAGTTTCCGTTGGAACAGTGAA |
| *Infg* | Interferon gamma | AGCGGCTGACTGAACTCAGATTGTAG | GTCACAGTTTTCAGCTGTATAGGG |
| *Il10* | Interleukin 10 | GCTCTTACTGACTGGCATGAG | CGCAGCTCTAGGAGCATGTG |
| *Ptgs2 (*COX2*)* | Prostaglandin-endoperoxide synthase 2 | TGACCCCCAAGGCTCAAATAT | TGAACCCAGGTCCTCGCTTA |

CD11c, cluster of differentiation 11c; COX2, ciclooxigenase 2; F4/80, macrophage marker; LXR, liver X receptor; MCP1, monocyte chimioattractant protein 1; TNF-α, tumor necrosis factor;
