# Supplementary material for: Functional Effects of EPS-Producing Bifidobacterium Administration on Energy Metabolic Alterations of Diet-Induced Obese Mice
Source: Front Microbiol. 2019 Aug 7;10:1809. doi: 10.3389/fmicb.2019.01809 (PMC6693475; doi:10.3389/fmicb.2019.01809)
Supplement: Supplementary file 2 [file Table_2.docx]

**Supplementary Table 2**: Gene expression levels of key inflammatory markers in adipose tissue and different sections of the gut and several parameters related to gut homeostasis in ileum.

|  | **CT** | **HF** | **HF-B** |
| --- | --- | --- | --- |
| **VAT** | | | |
| CCL2 | 1.00±0.14 | 0.60±0.08 | 0.88±0.14 |
| CD68 | 1.00±0.09 | 1.03±0.20 | 1.08±0.13 |
| F4/80 | 1.00±0.13 | 0.88±0.22 | 0.96±0.15 |
| CD11c | 1.00±0.17 | 0.79±0.17 | 1.03±0.12 |
| TNF | 1.00±0.19 | 1.11±0.28 | 1.42±0.15 |
| **SAT** | | | |
| CCL2 | 1.00±0.22 | 0.949±0.10 | 1.01±0.17 |
| CD68 | 1.00±0.21 | 0.980±0.10 | 1.01±0.13 |
| F4/80 | 1.00±0.28 | 0.801±0.09 | 0.83±0.14 |
| CD11c | 1.00±0.16 | 1.751±0.27 | 1.62±0.21 |
| TNF | 1.00±0.17 | 2.037±0.35 | 1.77±0.22 |
| **JEJUNUM** | | | |
| CCL2 | 1.00±0.14 | 0.73±0.05 | 0.78±0.06 |
| IL1B | 1.00±0.12 | 0.74±0.07 | 0.79±0.00 |
| IL6 | 1.00±0.15 | 0.64±0.12 | 0.95±0.13 |
| TNF | 1.00±0.13 | 0.94±0.08 | 0.90±0.11 |
| F4/80 | 1.00±0.08 | 0.90±0.03 | 0.99±0.04 |
| COX2 | 1.00±0.17 | 0.81±0.05 | 0.94±0.07 |
| **ILEUM** | | | |
| CCL2 | 1.00±0.17^a^ | 1.93±0.42^b^ | 1.01±0.07^ab^ |
| IL1B | 1.00±0.13 | 1.47±0.22 | 1.01±0.09 |
| IL6 | 1.00±0.18 | 0.76±0.15 | 0.65±0.15 |
| TNF | 1.00±0.09^a^ | 2.76±0.56^b^ | 1.51±0.22^ab^ |
| F4/80 | 1.00±0.27 | 0.92±0.11 | 0.73±0.16 |
| COX2 | 1.00±0.19 | 1.18±0.24 | 0.77±0.07 |
| IL10 | 1.00±0.17 | 1.08±0.11 | 1.16±0.10 |
| INFG | 1.00±0.26 | 1.30±0.15 | 0.93±0.13 |
| TJP1 | 1.00±0.24 | 0.93±0.17 | 0.89±0.20 |
| OCLN | 1.00±0.33 | 0.73±0.16 | 0.80±0.24 |
| MUC2 | 1.00±0.14 | 1.17±0.10 | 1.16±0.10 |
| REG3G | 1.00±0.15 | 0.63±0.19 | 0.80±0.20 |
| **COLON** | | | |
| CCL2 | 1.00±0.12 | 0.82±0.01 | 1.11±0.21 |
| IL1B | 1.00±0.14 | 1.05±0.09 | 0.93±0.08 |
| IL6 | 1.00±0.20 | 0.83±0.12 | 0.78±0.12 |
| TNF | 1.00±0.24 | 0.79±0.01 | 0.85±0.01 |
| F4/80 | 1.00±0.22 | 1.02±0.17 | 1.13±0.18 |
| COX2 | 1.00±0.14 | 1.20±0.14 | 1.49±0.36 |

Gene expression levels of key inflammatory markers in adipose tissue and different sections of the gut and several parameters related to gut homeostasis in ileum after a short-term high fat diet (HFD). Mice fed a control diet and delivery vehicle-skimmed milk in drinking water (CT), mice fed a HFD and delivery vehicle-skimmed milk in drinking water (HF) and mice fed a HFD supplemented with a suspension of 5x10^8^ cfu/mouse/day of *B. animalis* IPLA R1 strain in skimmed milk (HF-B) added to the drinking water. Data are expressed as the mean± SEM. VAT, visceral adipose tissue; SAT, subcutaneous adipose tissue; CCL2C-C motif chemokine ligand 2; CD68, cluster of differentiation 68, F4/80, macrophage marker; CD11c, cluster of differentiation 11c; TNF, tumor necrosis factor; IL1B, Interleukin 1β; IL6, interleukin 6; COX2, ciclooxigenasa 2; IL10, interleukin 10; INFG, interferon gamma; TJP1, tight junction protein 1; OCLN, occludine; MUC2, mucin 2; REG3G, regenerating family member 3 gamma.
