# Supplementary material for: Functional Effects of EPS-Producing Bifidobacterium Administration on Energy Metabolic Alterations of Diet-Induced Obese Mice
Source: Front Microbiol. 2019 Aug 7;10:1809. doi: 10.3389/fmicb.2019.01809 (PMC6693475; doi:10.3389/fmicb.2019.01809)
Supplement: Supplementary file 3 [file Table_3.docx]

**Supplementary Table 3**: Liver fatty acid profile.

|  | **CT** | **HF** | **HF-B** |
| --- | --- | --- | --- |
| **Total fatty acid identified** | 44.48±3.52 | 45.95±6.17 | 35.97±2.61 |
| **SFA** | 13.88±1.04 | 15.52±1.79 | 12.22±0.75 |
| C16:0 | 10.26±0.81 | 10.42±1.52 | 8.06±0.60 |
| C22:0 | 0.02±0.00 | 0.02±0.00 | 0.02±0.00 |
| **MUFA** | 11.96±1.16 | 12.50±2.96 | 8.39±1.10 |
| C16:1 (trans-9) | 0.01±0.00 | 0.00±0.00 | 0.00±0.00 |
| C18:1 (trans-9) | 0.02±0.00 | 0.03±0.00 | 0.018±0.00 |
| C18:1 (trans-10) | 0.00±0.00 | 0.02±0.00 | 0.02±0.00 |
| C18:1 (trans-11). | 0.02±0.00 | 0.02±0.00 | 0.02±0.00 |
| C18:1 (cis-9) | 9.26±0.88 | 10.79±2.51 | 7.30±0.98 |
| **PUFA** | 1.86±1.42 | 17.93±1.47 | 15.35±0.82 |
| C18:2 (n-6) | 10.62±0.90 | 9.15±1.32 | 7.17±0.54 |
| C18:2 (cis-9, trans-11) : | 0.03±0.00 | 0.03±0.01 | 0.02±0.00 |
| C18:2 (cis-9, cis-11) | 0.01±0.01 | 0.01±0.00 | 0.01±0.00 |
| C18:2 (trans-11, trans-13) | 0.01±0.00 | 0.01±0.00 | 0.01±0.00 |
| C18:2 (trans-9, trans-11) | 0.01±0.00 | 0.01±0.00 | 0.01±0.00 |
| C22:5 (n-3) | 0.14±0.01 | 0.13±0.01 | 0.19±0.01 |
| C22:6 (n-3) | 2.88±0.23 | 2.94±0.12 | 2.83±0.12 |
| C18:1 C9/ C16:0 | 2.61±0.17 | 2.09±0.38 | 1.79±0.22 |

Non-statistically significant (p>0.05) liver fatty acid profiles and desaturation ratio after a short-term high fat diet (HFD). Mice fed a control diet and delivery vehicle-skimmed milk in drinking water (CT), mice fed a HFD and delivery vehicle-skimmed milk in drinking water (HF) and mice fed a HFD supplemented with a suspension of 5x10^8^ ufc/mouse/day of *B. animalis* IPLA R1 strain in skimmed milk (HF-B) added to the drinking water. Data are expressed as the mean± SEM.
